# Promotors and barriers to the implementation and adoption of assistive technology and telecare for people with dementia and their caregivers: a systematic review of the literature

**DOI:** 10.1186/s12913-022-08968-2

**Published:** 2022-12-23

**Authors:** Lydia D. Boyle, Bettina S. Husebo, Maarja Vislapuu

**Affiliations:** 1 Department of Global Public Health and Primary Care, Centre for Elderly and Nursing Home Medicine, University of Bergen, Årstadveien 17, 5009 Bergen, Norway; 2 Department of Global Public Health and Primary Care, Centre for International Health, University of Bergen, Årstadveien 17, 5009 Bergen, Norway; 3 Neuro-SysMed Center, Department of Global Public Health and Primary Care, University of Bergen, Bergen, Norge

**Keywords:** Dementia, Assistive technology, Telecare, Caregivers, Implementation, Barriers, Promotors

## Abstract

**Background:**

One of the most pressing issues in our society is the provision of proper care and treatment for the growing global health challenge of ageing. Assistive Technology and Telecare (ATT) is a key component in facilitation of safer, longer, and independent living for people with dementia (PwD) and has the potential to extend valuable care and support for caregivers globally. The objective of this study was to identify promotors and barriers to implementation and adoption of ATT for PwD and their informal (family and friends) and formal (healthcare professionals) caregivers.

**Methods:**

Five databases Medline (Ovid), CINAHL, Web of Science, APA PsycINFO and EMBASE were searched. PRISMA guidelines have been used to guide all processes and results. Retrieved studies were qualitative, mixed-method and quantitative, screened using Rayyan and overall quality assessed using Critical Appraisal Skills Programme (CASP) and Mixed Methods Assessment Tool (MMAT). Certainty of evidence was assessed using Grading of Recommendations Assessment, Development and Evaluation (GRADE) criteria and assigned within categories of high, moderate, or low. NVivo was used for synthesis and analysis of article content. A narrative synthesis combines the study findings.

**Results:**

Thirty studies (7 quantitative, 19 qualitative and 4 mixed methods) met the inclusion criteria. Identified primary promotors for the implementation and adoption of ATT were: personalized training and co-designed solutions, safety for the PwD, involvement of all relevant stakeholders, ease of use and support, and cultural relevance. Main barriers for the implementation and adoption of ATT included: unintended adverse consequences, timing and disease progress, technology anxiety, system failures, digital divide, and lack of access to or knowledge of available ATT.

**Conclusion:**

The most crucial elements for the adoption of ATT in the future will be a focus on co-design, improved involvement of relevant stakeholders, and the adaptability (tailoring related to context) of ATT solutions over time (disease process).

**Supplementary Information:**

The online version contains supplementary material available at 10.1186/s12913-022-08968-2.

## Background

There are approximately 57.4 million people living with dementia (PwD) globally [1]. According to Alzheimer’s Disease International, numbers of dementia are growing fastest in China, South Asia, India and western Pacific countries [2]. The Lancet’s Global Burden of Disease Study estimates that global prevalence will increase by an average of 166% by 2050 [1].

PwD are faced with a multitude of complex symptoms including, but not limited to, memory deficits, behavioral and psychological symptoms of dementia (BPSD), depression, and pain [3–5]. This results in increased caregiver burden in formal (health care professionals) and informal (friends and family) caregivers [6–10]. Other health-related consequences for informal carers include increased levels of depression, anxiety, and low self-perceived physical health [7, 8]. Similarly, formal caregivers experience increased stress, psychological, physical and social distress and burnout. The result is a loss of productivity in the workforce, increased sick-leave and hospitalization, and systemic economic burden within healthcare systems [6, 9].

A recent Lancet Commission Report explored dying in the 21st century and the “value of death” [11]. The commission was created to address the changes which have occurred over recent generations concerning how people die. The authors argue that radical change is needed with greater demand for novel healthcare solutions [11]. ATT is broad in definition and the healthcare digital revolution, most recently fueled by COVID-19, has seen exponential growth over the last decade [12]. Telehealth, e-Health, telemedicine, telecare, assistive technology, welfare technology, digital therapeutics, and information and communication technology are commonly used interchangeably within the literature [13]. For further purposes of this paper, we will consider these terms to include any digital tool or technology that is used as a means of remote healthcare service for the PwD or caregiver. These can include videoconference evaluation or treatment, wearables, sensors, smart homes, and digital devices (e.g., smartphone, tablet) which expand homebound services and support for PwD and caregivers (formal and informal). Adoption and implementation are terms that are also frequently used interchangeably. Implementation is generally defined as “the process of putting a decision or plan into effect; execution” [12]. For purposes of this systematic review, implementation can be defined as the process of putting ATT in place (home or care home) with the goal of eventual adoption and habitual daily use of ATT in a “real world” setting. Adoption should be understood as an evaluated consequence and potential result of implementation [14]. Simply, adoption can be seen as putting a technology to habitual use after implementation, while implementation is at the point when the technology becomes available [14].

A 2020 systematic review synthesizing evidence on sensor technology for PwD found that sensors are most frequently used to monitor BPSD such as sleep disturbances, agitation, and wandering [15]. Internet of Things (IoT) technology is a fairly new concept of in-home sensor monitoring that offers promising options for home-dwelling PwD [16]. IoT technology can include wearables, biometric sensors, smartphones, apps, smart home ambient sensors, environmental sensing, indoor positioning sensors, microphones, wearable and mounted cameras [16]. Wearables, such as FitBit, are another popular IoT on the market which is being used to detect and monitor levels of activity and biomarkers such as heart rate, sleep patterns, and blood pressure [16, 17]. Smart home design incorporates sensing technology, wearables, smart phones, and integrated assistive devices that can include cameras, touch screens and voice technology, to increase safety and independence for PwD living at home. In existing literature, terminology related to smart homes has evolved and is often referred to as “unobtrusive in-home health monitoring” [18]. Robots as a means for social care, communication and intervention for PwD are referred to as socially assistive robots (SARs) such as “petbots” (e.g., Paro) [19].

Systematic reviews recognize the gap of quality implementation research on ATT interventions. Christie et al. (2018) identifies a mismatch between research being conducted on eHealth interventions and the use of implementation frameworks and encourage better focus on end user involvement (informal caregiver) [20]. Peek et al. (2014) demonstrates scarcity of research on acceptance of ATT for home-dwelling PwD [21]. Furthermore, previous studies ask for inclusion of broader contextual factors, such as sociocultural, time-restraints and organizational constructs of implementation [20, 22]. The purpose of this systematic review is to identify promotors and barriers to implementation and adoption of ATT for PwD and their informal (family and friends) and formal (healthcare professionals) caregivers and (1) to identify promotors and barriers that are common across research settings (home and institution environments); (2) to identify and analyze common themes within the literature; (3) to propose novel implementation strategies which may improve implementation and adoption of ATT globally.

## Methods

This systematic review presents a synthesis of previous research on the promotors and barriers for implementation of ATT in PwD and their informal and formal caregivers. This review followed the recommendations established by Snyder in 2019 to ensure quality of content and results [23]. PRISMA guidelines were used to ensure proper inclusion categories and quality, and transparent reporting [24, 25]. The study is registered in PROSPERO 25th of February 2021 [CRD42021239448]. Rayyan QCRI software was utilized for screening of all literature. To reduce the risk of bias and assure overall quality, the Critical Appraisal Skills Programme (CASP) and the Mixed Methods Appraisal Tool (MMAT) were utilized [26, 27]. NVivo software was used for support and visualization of the analysis process and to pull themes from the qualitative literature.

Certainty of evidence was assessed using Grading of Recommendations Assessment, Development and Evaluation (GRADE) criteria and based upon answers to specific questions in the CASP and MMAT assessments for quality and bias. Questions were assigned a 0–1 rating (1-yes, 0-can’t tell and no) and categorized as certain [1] or uncertain (0). The questions were further analyzed by dividing the total number of «certain» or «yes» answers [1] by the total amount of questions on the assessment and given a percentage (0-100%) depending on this rating. Certainty of evidence is defined in the Tables 1 and 2 for each included article as high (80–100%), moderate (50–79%) or low (0–49%). Further summary of assigned quality percentages can be found in Additional file 1.

**Table 1:** Barriers and promotors, quantitative literature, *N* = 7

**Author, country, year**	**n**	**Design**	**Certainty of the evidence (GRADE)**	**Assistive Technology**	**Barriers**	**Promotors**
Asghar, I., et al., Pakistan (2019) [28]	327	Cross-sectional	Moderate (55%)	Mobility Support Cognitive Games Reminder or Prompter Social Application Leisure Support	Operational support Physical Support Psychological support Social Support Cultural match Affordability	AT effectiveness: AT psychological support & AT social support Physical support AT retention: Reduced external help, AT travel help, AT culture match
Dai, B. Z., et al., Sub Saharan Africa (2020) [29]	350	Cross-sectional	Moderate (64%)	Wearables	Technology anxiety Resistance to change Malfunction of ATT Costs	Subsidized costs Training and clearly communicated benefits of use social influence facilitating conditions (context, cultural, environment) effort expectancy
Jarvis et al., Australia, 2017 [30]	85	Cross-sectional	Moderate (55%)	Way-finding technology	Limited awareness of how ATT is used for support PwD Limited knowledge of available ATT Lack of time and information Costs Difficulty learning new skills	N/A
Lauriks et al., Netherlands, 2020 [31]	54 25	Pilot study, RCT	High (82%)	Alerts, lighting and design (non-obstruction)	Malfunctions, errors Fidelity	N/A
Coco et al., Finland and Japan, 2018 [32]	286	Cross-sectional	Moderate (55%)	Robots	Decreased QOL Fear of job loss Lack of trust usefulness of robot to conduct tasks beyond simple intervention	N/A
Dugstad, J., et al., Norway (2019) [33]	67 172 23	Longitudinal case study	Moderate (73%)	Digital night surveillance interventionIot	N/A	Development of clear Pre-implementation and Implementation strategies including: Managing risks Reflection Co-creation Tailored training Involving all stakeholders Culture match Common language Continuous evaluation Developing new roles Realizing benefits Compatibility with existing services Scaling up gradually Facilitate dialog Establish a team of champions Promote co-creation through workshops
Øksnebjerg, L. et al., Denmark (2020) [34]	19	Pilot study	Moderate (64%)	React app	N/A	Identification of goals prior to implementation Ease of use Individual and group-based activities

*N* number of studies, *n* number of participants included in the study, *N/A* not applicable

**Table 2:** Barriers and promotors, qualitative and mixed methods literature, *N* = 23

Author, country, year	n	Design	Certainty of the evidence (GRADE)	Assistive Technology	Barriers	Promotors
Arntzen, C., et al., Norway, 2016 [35]	12	Phenomenological study	Moderate (60%)	Various ATT	Habitual practices Negative emotions Poor design Not adaptable Not engaging the carer Complexity of ATT	Fit with habitual behaviors Culture Trust user-friendly Adaptability
Arthanat, S., et al., USA, 2020 [36]	8	Focus group interviews	Low (40%)	Socially assistive robot (SAR)	Technology anxiety Effort expectancy Structure and design of the home Value and worth Digital Divide System failures Dual burden	Trust (fidelity) Personalized training Adaptability (tailoring) Engaging the care recipient Humanoid features
Egan, K. J. And A. M. Pot, USA, Australia, Canada, China, India, Japan, Netherlands, United Kingdom, 2016 [37]	66	Qualitative, Focus group interviews	High (80%)	Varied ATT	Stigma Poor accessibility Not accounting for disease progression	Raise awareness Affordability Integrate with existing services Increase collaborative approaches including the PwD
Evans et al., UK, 2017 [38]	48	Mixed methods (qualitative, self-administered questionnaires)	Moderate (71%)	Ipads - games, memoirs, video conference	Benefits and Barriers: Ease of use Convenience and Flexibility Portability Cost	N/A
Faeo, S.E. et al., Norway, 2020 [39]	12	Qualitative, exploratory	Moderate (70%)	Various ATT	Safety with side-effects (unintended consequences) unmet expectations for volunteerism diversity of care and services	A way to broaden PwD everyday environment Ability to have more freedom - walking, out from house Maintained dignity
Fange, A.M., Norway, 2020 [40]	9 21	Qualitative, semi-structured interviews	High (90%)	Sensors	Not having a clear understand of the benefits of ATT Unreliable technology Not fitting into habits Lack of control over an installed device Ethical issues - privacy	Safety for the PwD ATT as a support to make life easier Complemented established care
Gibson, et al., UK, 2015 [41]	13 26	Qualitative, Semi-strctured interviews	High (90%)	DIY ATT, off the shelf solutions	Too little too late from formal care (ATT) Cost	Role of the caregiver as facilitator Easily integrated with current habits/routines
Gibson, et al., UK, 2018 [42]	13 26	Semi-structured interviews	High (80%)	DIY ATT, off the shelf solutions	Inaccessibility Cost No information about technology for PwD “Crisis model” of implementation	Ability to incorporate into habitual practices Informal caregivers as facilitators and bricoleur Off-the-shelf solutions (accessibility and cost)
Hall A. et al., UK, 2017 [43]	36	Multiple-case study with qualitative methods	Moderate (70%)	Sensors, Memory aides	Key stakeholders not involved in implementation process Limited understanding from stakeholders regarding benefits and challenges of ATT	Enhanced safety Personalized training for staff & caregivers
Heuvel et al., UK, 2012 [44]	25	Qualitative, Focus group interviews	High (90%)	Various ATT	Lack of information unknown benefits of use	N/A
Holthe, T. et al., Norway, 2020 [45]	24	Qualitative, Focus group interviews	High (100%)	Various ATT	Unsystematic approaches Contested responsibility Citizen capabilities	Knowledge and training User friendliness
Holthe, T. et al., Norway, 2018 [46]	13	Qualitative, repeated semi-structured interviews	High (100%)	Various ATT	Waiting times Lack of information from public services Untimely information about ATT	Simply designed ATT Committed caregiver Need based provision Incorporation into habitual routines
Ienca et al., Switzerland Germany Italy, 2018 [47]	17	Open-ended qualitative interviews	High (90%)	Various ATT	Mismatch between patients’ needs and ATT Technical limitations Translational problems	See barriers
Kerssens et al., USA, 2015 [48]	7	Feasability study	Moderate (70%)	The Companion - touch screen with Psychosocial interacts for PwD	Not offering a feature counted on Caregivers ignoring or muting shows Recipients ignoring interventions Not having enough time Unwillingness to share experiences Unmet expectation	Relaxation Enjoyment of life Reminisce
Lindqvist et al., Sweden, 2013 [49]	17	Qualitative, semi- structured interviews	High (90%)	Various ATT	N/A	Trust for the ATT Perceived capacity for use Fitting into routines Pre-planning for a decision on which ATT was most appropriate
Lindqvist et al., Sweden, 2015 [50]	14 14	Qualitative, semi-structured interviews	High (90%)	Various - based on interviews with PwD and caregivers	Out of sight-out of mind Non-relevant info Professionals needed for updating features Small buttons Settings easily manipulated by mistake No instructions or feedback	Visibility of the ATT Visualized reminders Customizable features (user) Reminders delivered to mobile phone Personalized buttons Feedback and guidance on display
Mehrabian et al., France, 2015 [51]	92	Mixed methods (semi-structured interviews, self-administered questionnaires)	Moderate (53%)	Various ATT	Complexity Expectation vs. reality Perceptions of need by the caregiver Technology anxiety Costs Limited access to internet in the homes	Security and safety for the user Assisting in case of emergency Enable cognitive stimulation Reminders for meds Improvement in day-to-day living
Niemeijer, A. R. et al., Netherlands, 2014 [52]	43 28	Qualitative, ethnographic field study	High (90%)	Surveillance technology	False alarms Alarm fatigue Not using the technology to full potential Forgetting to take devices off Perception of staff	Vision of safe autonomy Informing of participants (risks and benefits) Instructions and training of staff Willingness to use new technology
Pino et al., France, 2015 [53]	25 7	Mixed method, (focus group interviews, self-administered questionnaires)	Low (41%)	SARs	Negative impact on autonomy Size of SAR Privacy concerns Fear of robots replacing humans/jobs Suitability for level of dementia Negative attitudes Generational gap Perceived usefulness Fear of the future	Cognitive support Communication and companionship Safety and healthcare use Supports independent living Alleviates caregiver stress
Snyder et al., USA, 2020 [54]	4	Qualitative, phenomenological study	High (90%)	Remote monitoring technology	Lack of technical ability Perception of technology as confusing or unclear Ease of use Not tailored to needs Lack of knowledge of benefits of use Ethical issues	Caregiver peace of mind better communication with pwd caregiver confidence caregiver and care recipient independence
Steils et al., UK, 2021 [55]	114	Mixed methods, (semi-structured interviews, case studies, self-administered questionnaires)	High (88%)	Various ATT	Lack of information unknown benefits of use carers level of knowledge of technology	Tailored solutions Involvment of carers
Thorpe et al., Denmark, 2016 [56]	10	Feasibility study	High (80%)	Sony smartwatch 3 and Sony Xperia E4	Navigation and emergency support	Scheduling features Familiar design Personalization
Yaddadin et al., Canada, 2020 [57]	24	Qualitative, focus group interviews	Moderate (50%)	Various ATT	Complexity of ATT Difficulty adapting Requires a large number of resources (time and costs) Resistance to the use of a technological aid	Learning potential Interdisciplinary collaboration (including the family) Experience Varied features of COOK

Table legend: *N *number of studies, *n *number of participants included in the study, *N/A *not applicable

### Search strategy

We searched the following five databases for relevant literature: Medline (Ovid), CINAHL, Web of Science, APA PsycINFO and EMBASE. Keywords included MESH terms and phrases synonymous as follows: “dementia” AND “assistive technology” OR “telecare” OR “telemedicine” OR “e-health” AND “implementation” OR “barriers” OR “promoters” OR “facilitators”. Search strategy and key terms were further developed using these resources (Additional file 2).

#### Inclusion and exclusion criteria

Studies were included if they met all of the following criteria: (1) uses ATT or other defined technology-based intervention to deliver an individually tailored solution to PwD and/or their formal or informal caregivers, (2) reports findings or thoughts as to the implementation of these interventions within the abstract or text and/or barriers to implementation of assistive technologies, (3) PwD are classified by a health professional as having mild-severe dementia based on a validated cognitive outcome measure such as the Mini-Mental Status Examination (MMSE), Functional Assessment Staging Tool (FAST) or Clinical Dementia Rating scale (CDR), (4) publications from 2011 to 2021 and, (5) global publications, written in English. Studies prior to 2011 were not included as prior research may not be as applicable to integration and implementation into current healthcare systems. We take consideration for the increase in technological development and use since the beginning of 2019 fueled by the pandemic (COVID-19).

Studies were excluded if they met any of the following criteria: (1) technology related specifically to COVID-19 interventions, (2) report findings solely relating to general technology rather than the PwD and/or their formal or informal caregiver, (3) findings that do not directly or indirectly address the topic of implementation of and/or barriers to implementation of technology-based interventions, (4) interventions related to comorbidities and other diagnoses such as stroke, diabetes, HIV or heart disease, (5) literature regarding specific categories of ATT such as wheelchairs or occupational therapy devices for activities of daily living, (6) opinion papers, literature reviews, theoretical papers, study protocols, and conference abstracts.

### Article screening and data extraction

After removal of duplicates and based on Rayyan, two authors (LB and MV) screened manuscripts based upon title and abstract. Potentially relevant studies were assessed for eligibility by all authors by evaluating the inclusion and exclusion criteria on the full-text manuscripts. Reference lists of manuscripts and reviews were screened to identify additional relevant publications. An excel form was used for initial data extraction and the following key elements were extracted from each article: study design, country, focus of study, population and study setting. Furthermore, topic specific issues such as the type of ATT included and barriers and promotors, were extracted for each article. We further identified arching themes and key topics from this information. The final selection of included publications was by consensus among all authors.

## Results

**Fig. 1:**
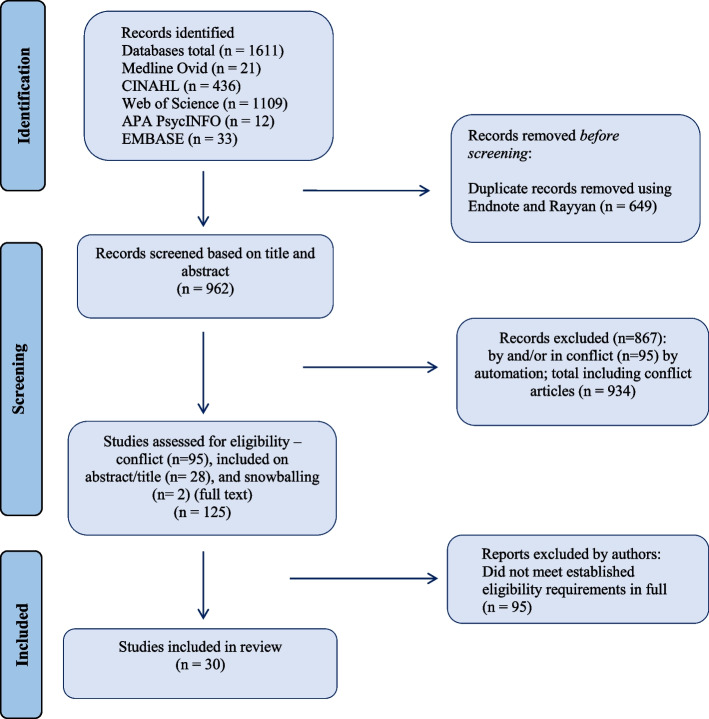
Flow diagram of study selection process

The initial search generated at total of 1,611 potential publications, of which 30 papers were identified as relevant for inclusion (Fig. 1). Of these, 7 were quantitative (Table 1), 19 qualitative and 4 mixed methods (Table 2). Two of the included articles were added using snowballing techniques. The review includes literature representing five continents and sixty-five countries globally. 94% of the publications are from high-income countries. Quality assessment was performed for each included article using CASP (qualitative and quantitative) and MMAT (mixed-methods) (Additional File 3) [26, 27].

### Promotors

#### Personalized (tailored) training and education

The top promotor to implementation and adoption of ATT for PwD and their caregivers (formal and informal) was tailored training and education for all stakeholders involved in the implementation [32–34, 36, 38, 41, 45, 57]. Specific examples within the literature were university sponsored courses or workshops, online-learning, demonstrations of the technology for the family, hands-on-practice with the ATT prior to implementation, support networks for post-implementation trouble-shooting and designated “super-users” at various levels for continued support [32–34, 36, 38, 41, 45, 57]. In several of the included studies, education was seen to play a crucial role in the acceptance of the new technology and in establishing positive attitudes towards its reliability [32, 33, 36].

A cross-sectional study by Coco et al. (2018) compared survey findings regarding the acceptability of SARs among 286 healthcare workers in nursing homes in Finland and Japan [32]. They conclude that management plays a vital role in education efforts for personnel and that training and education is crucial for acceptance of innovation, understanding of benefits for ATT, diminishing fears and negative thoughts, and in changing attitudes which could detour adoption. This was especially emphasized concerning situations where ATT is being implemented in varied cultural contexts [32].

Dugstad et al. (2019) conducted a 4-year longitudinal case study of the implementation of monitoring technology in 67 Norwegian nursing homes [33]. They concluded that personalized training should be initiated for a variety of stakeholders skills and the development of a common “language” to bridge gaps between professionals and stakeholders [33]. These stakeholders include multiple industries and levels of care. For example, governing officials within the municipality, management of private and public health institutions and varying layers of their staff, service providers such as home health, IT and axillary services within the home (cleaning staff, etc.), physicians and specialists, caregivers (formal and informal), developers and providers of ATT services, and the PwD.

#### Safety for the PwD

The safety and wellbeing for PwD often superseded ethical considerations in regards to the decision for implementation of ATT [28, 36, 40, 45, 47, 58]. Dugstad et al. (2019) found that ATT implementation within nursing homes facilities fostered a “safety culture”, which bolstered the feeling of “saving lives” [33]. Findings suggest that not only PwD and their informal caregivers may hold this belief, but that this also occurs at organizational levels within healthcare facilities [33].

A qualitative study by Fange et al. (2020) [40] explored the experiences, needs and benefits with using sensor-based technologies for safety and independence in the homes of PwD and their family members (*n* = 30) [40]. Participants were recruited from the TECH@HOME project (*n* = 640) (2016–2019) in Sweden and found that ATT was viewed as a support to make life easier and safer [58]. Both studies found that there is a continuous negotiation between safety and privacy for PwD and informal caregivers especially, when it comes to continuously assessing informed consent by participants to use the technology in their home [40, 58].

#### Involvement of stakeholders

Many of the included studies concluded that involvement of appropriate stakeholders promoted successful implementation and adoption of ATT [33, 35, 37, 40–42, 46, 59]. Examples of these stakeholders were the informal caregiver or PwD [33, 35, 40–42, 46, 59], key personnel (taking consideration of shift changes) [33], key IT personnel at the municipality level [59], management at the healthcare facility [33], other non-IT personnel that had indirect impact on implementation such as janitors and support staff [33], and home health personnel [46].

KJ Egan and AM Pot (2016) utilized multinational (United States, Australia, Canada, Japan, Netherlands, United Kingdom, China, India) focus groups and a variety of stakeholders (PwD, representatives working in industry, academic researchers, regulators, research funders, policy makers and formal and informal care providers) to identify six key elements for the future development of ATT: (1) raise awareness and reduce stigma, (2) improve accessibility and affordability, (3) to integrate with existing services, (4) to increase collaborative approaches and make PwD a part of the process, (5) to account for disease progression and (6) to facilitate and develop implementation of innovative ATT [37]. The study concluded that “there is an overriding imperative for a systematic, coordinated multistakeholder approach with the needs of PwD and their caregivers (informal and formal) as the centerpiece” [37].

Much of the included research involved PwD living within nursing homes  [14, 32, 33, 38, 45, 47, 50, 53, 60], however a qualitative study (survey) including Australian occupational therapist (*n* = 87) by Jarvis et al. (2017) explored the prescription of ATT for home-dwelling PwD [30]. 51% of the participants did not prescribe ATT for PwD with wandering tendencies because of: limited knowledge about the type of technology available, limited resources available to provide ATT, concern about the client and their informal caregivers ability to meet the costs of the ATT and difficulty learning new skills [30]. Another survey by Steils et al. (2021) looked at the perspectives of council telecare managers and stakeholders (*n* = 114) in the UK concerning informal caregiver involvement in telecare provision [59]. They found that a promotor to the usefulness and adoption of ATT was proper provision of information and knowledge and suggested improved training, provision for self-installation and better support packages for informal caregivers post-implementation [59]. Generalization from the studies conducted within nursing homes cannot fully be made, however they can be viewed as a core road-map for home-dwelling strategies. This also raises consideration for future research topics concerning implementation for home-dwelling PwD.

#### Ease of use

The ease of use of the ATT is considered a significant promotor for implementation and adoption. The simplest of technology was often the most likely candidate to be successfully incorporated into daily habits of PwD and both formal and informal caregivers [37, 38, 41, 42]. These technologies were seen to enhance established daily routines and were described as flexible, convenient, simple, portable, clear in instructions, and with enlarged font size [38, 47, 50, 53, 57].

Evans et al. (2017) introduced iPads into 63 UK nursing homes and investigated the experiences and potential benefits in PwD and their formal and informal caregivers [38]. The ease of use of the iPad, integration into everyday activities, and different tasks were a key promotor for successful implementation and adoption. During the project, iPad utilization increased from 15 to 80% [38].

#### Cultural relevance

Differences in usefulness and acceptance of ATT were noted between cultural groups, therefore pushing cultural relevance forward as a primary influencer for promotion of implementation and adoption of ATT [29, 32, 33, 47, 50, 53, 61]. The term culture can constitute many definitions. Cultural differences addressed in this study include origin of study (country), spiritual and religious differences/beliefs, stigma surrounding diagnosis of dementia, language, and professional belief system/differences in communication and language (industry). The longitudinal case study by Dugstad et al. (2019) demonstrated that proper planning impacted the implementation process and established bonds between stakeholders leading to a common language between professional groups [33]. Ienca et al. (2018) investigated the need for common language from a multinational perspective (Switzerland, Germany, Italy) including health professionals and researchers (*n* = 17) [47]. They found that an intermediary platform could potentially bridge the gaps across relevant stakeholders (e.g., clinicians and tech-producers) [47].

A cross-sectional study by Coco et al. (2018) (*n* = 286) investigated the beliefs surrounding implementation of care robots in Finland and Japan and demonstrated larger acceptance for assistive robotics in Japan [32]. 40% of the Finnish respondents considered the SAR to be inhumane (compared to 8% in Japan) [32].

### Barriers

#### Unintended adverse consequences

Many of the examples stated within the literature include descriptions of negative technology related emotions from both the PwD and caregivers (informal and formal) alike. From the point of view of the PwD, failed attempts to use the ATT often caused feelings of incompetence, confusion, annoyance, and stress [28, 41, 45, 46, 50, 58]. The formal caregivers expressed a wide range of feelings associated with fear, which included fear of being replaced by the ATT, fear that the ATT dehumanized, increase loneliness or infantilized the PwD and fear for the safety of the PwD due to malfunctioning ATT [32, 53]. There were also feelings of fatigue, confusion, mistrust of the ATT and increased stress from the caregivers (formal and informal) [45, 54].

#### Timing of implementation and disease progression

Studies which addressed timeliness concurred that ATT should be given as an option in the earliest stages of diagnoses, and in some instances before diagnoses when the PwD is demonstrating early symptoms of dementia [34, 35, 37, 40–42, 46, 50, 54, 61, 62]. A qualitative study by Arntzen et al. (2016) looked at successful incorporation of ATT for 26 younger PwD and family caregivers and emphasize the importance of timely, tailored interventions to meet the cognitive conditions [35]. The study found that the introduction of ATT was most successful when introduced early and corresponding to daily routines [35].

A qualitative study by Gibson et al. (2019) included 39 PwD and informal caregivers and found ATT being introduced too late and introduced post-crisis (e.g., after a fall or wandering incident) [42]. The development of subsequent strategies to emphasize a proactive vs. reactive goal for ATT adoption in this setting are strongly recommended.

#### Technology anxiety

Fange et al. reported on using sensor technology to foster independence and safety for PwD, utilizing participants (*n* = 30) [40] and data from the larger RCT TECH@HOME trial (*n* = 640)[58]. The study, using an inductive, qualitative design and semi-structured interviews, found that some healthcare workers seemed to be afraid and distressed by new technology and at times unintentionally tampered with hardware without knowing what they were doing or how to fix it [40]. Technology anxiety can be reduced and addressed by deploying specific strategies for dialog with both the PwD and their caregivers (formal and informal) [40, 63].

Informal caregivers involvement in telecare provision from the perspective of council telecare managers and stakeholders was studied by Steils et al. [59]. The three-staged, mixed-method design included interviews with telecare managers (*n* = 27), case studies (*n* = 21) and a survey of councils (*n* = 114) [59]. The results of the study reported on reasons why formal telecare had been decommissioned at the request of the recipient or informal caregiver. One main finding was that this occurred because the informal caregiver felt the ATT had become invasive and caused anxiety to the older person, and/or that the PwD was unable to reliably operate the device. This had a direct negative impact upon the informal caregivers [59].

#### System failures, errors, lack of connectivity

Burdens such as system failures, various errors in programming and issues with connectivity have the potential to “tip the scale” in favor of rejection of ATT. In some instances, failures in initial processes and planning for the implementation were reason for eventual system failure, and overall rejection of the ATT. Dugstad et al. (2019) gives an example of this in their longitudinal study (*n* = 67) conducted in Norway, which investigated co-creation and the implementation of monitoring technology in residential care for PwD, and refers to an integral period they call “pre-implementation” [33]. Here the authors found that important factors in this pre-planning phase were missing in 7 of 8 Norwegian municipalities included within the study. These included basic elements such as initial risk assessments, patient safety assessments, compatibility assessment between current and future technology, security assessments and involvement of all required key stakeholders [33]. The result was that inevitably instability and error occurred, creating an array of frustration, poor service delivery, security risks to the PwD and instability in the overall infrastructure at the municipality level [33]. The study concluded that reliability of the technology was crucial, and that IT infrastructure and mobile network instability were the major persistent barriers to implementing the monitoring system [33].

Poor quality of hardware and software was seen as a risk factor that could harm the overall reputation of the ATT market [47]. A 2018 qualitative study by Ienca et al. (*n* = 17) investigated technology for psychogeriatric care using interviews in a multinational context (Switzerland, Germany and Italy) and looked at health professionals and researchers views on intelligent ATT [47]. One viewpoint taken from the interviews was that the ATT market included numerous poorly designed, clinically ineffective and insufficiently validated devices [47].

#### Digital literacy

Digital literacies or competences can be described as the knowledge, skills and dispositions needed in order to utilize ATT [64]. As the complexity of available and emerging technology increases, the concept of digital literacies presents as a challenge and is a highly debated topic in the fields of healthcare, education and research currently [64]. When specifically applied to people with cognitive impairment, competency and understanding of topics such as ethics and sustainability of digital services also take center stage as these users are especially vulnerable [65]. Within the last decade there has been a push to standardize the approach to digital literacies. Some argue that universalization of digital literacy approaches can be problematic and that a better solution may be a cross-national, multidisciplinary blending of concepts [64].

#### Lack of access to or knowledge of ATT

Limited access to knowledge about the type of technologies available and limited resources available for the provision of ATT are a barrier to the implementation of ATT in various contexts [29]. One may assume that this context is referring to primarily LMIC settings. Although accessibility may fall into a larger category within the hierarchy of barriers, it is certainly not limited to LMIC. Accessibility limitations in mid-high level income countries still include lack of basic provision such as internet access (although to a lesser degree), but main access limitations here are due to lack of knowledge and organizational restraints [34, 38, 59, 66].

Dai et al. (2020) (*n* = 350) conducted a survey which looked at factors affecting the acceptance of wearable devices by PwD in English speaking countries within Sub-Saharan Africa, and found that limited access to ATT created hesitation by informal caregivers to encourage use for PwD [29]. High income countries defined accessibility differently. This included that the general physician and/or healthcare workers had not informed the PwD or informal caregiver about ATT as a part of the dementia care possibilities, policy restraints and a general lack of knowledge regarding available ATT by both formal and informal caregivers [29, 30, 45].

## Discussion

Investigation of the promotors and barriers to implementation and adoption of ATT for PwD and their caregivers (formal and informal) revealed five arching topics. These include tailored solutions and training, ethics, and safety for PwD, timeliness of intervention, cultural relevance, and improved strategies for implementation and future research. Knowledge surrounding these factors can shape how ATT is developed, researched, funded, and ultimately accepted within the market (by the end-user). Furthermore, we will discuss additional findings which include equity and fidelity, implementation frameworks and theories, and the concept of contamination. Implementation should be viewed as a “living” process in which there must be contingence and finite strategies for continued evaluation of the appropriateness and effectiveness of ATT for each user. Just as dementia and palliative care is defined along a spectrum, so should tailored ATT interventions be viewed. Sustainable implementation is well planned, continually evaluated, supported, and informed by the end-user. Understanding of the evolution and radical change which is potentially necessary at the municipality and government levels within the healthcare supply chain is essential to the future success of ATT implementation. Research conducted in areas of the world where dementia rates are predicted to grow the fastest over the next thirty-years is greatly warranted. Our findings within this systematic review should be a call to action for further research on this topic within LMICs.

### Tailored solutions & training

Tailored solutions and training with a multi-stakeholder approach is of utmost importance to the success of implemented ATT. Proper education for the healthcare teams which will provide continuation of care and support of ATT implementation beyond the policy levels should be a key strategy within the implementation plan. These stakeholders are often primary facilitators for the use and adoption of ATT. The pre-implementation phase is of critical importance in identifying all stakeholders and levels of tailored education needed. Healthcare workers have been found to be “late adapters” of new technology according to several studies [37, 40]. These studies indicated that the staff had insufficient knowledge of the ATT, inability to maintain the technology and at times were fearful of the ATT for various reasons including fear of job loss or replacement and having negative feelings towards the appropriateness of the ATT to maintaining dignity and safety for the PwD. A scoping review by D’Cruz et al. (2020) looked at tailored education of hospital patients with cognitive impairments [60]. Several barriers to tailored education were identified including time constraints by staff, use of jargon and lack of appropriate communication, and informal caregiver burden [60]. In regards to education for people with cognitive impairment, the authors suggest that programs should have variation in delivery of information (verbal and written, various time points, etc.) and should reflect individual cognition levels (re-tested often and systematically) and preferences of the client [60].

Education and training should involve a curriculum for improved knowledge of rights, ethics and safety concerning the provision of ATT. With regards to digital literacy for PwD and their formal or informal caregivers, a combined and flexible methodology would fit well with a co-design and patient centered strategy for improved future ATT implementation. This approach could allow for specialized conceptualization of ATT across globalized frontiers. Further development of novel tools like a multidimensional questionnaire for telehealth literacy screening, such as in the mixed-method study by Gillie et al. (2022) (*n* = 90), could be useful in determining levels of literacy and subsequent levels of training and education which are needed for successful implementation of ATT for home dwelling individuals [67]. Another avenue related to digital literacy is the concept of *dementia literacy*. Having a combined approach of novel education regarding disease process and ATT use, maintenance, and support can strengthen knowledge and awareness of dementia, decrease stigmas, and could intrigue interest for future ATT adoption throughout the spectrum of the disease. Another novel concept that was noted in several of the included studies was that of educating the PwD and informal caregiver to be able to educate others regarding the technology within their circle of influence [33, 34, 38, 54]. This concept incorporates aspects of ethical consideration for other auxiliary and support staff in the home, for example with use of smart home monitoring technologies, that may require general understanding and knowledge of the prescribed technology.

### Ethics & safety

The introduction of ATT often raises ethical considerations [68]. One interesting revelation within the included literature was that in many cases the PwD and informal caregivers considered the feeling of “safety” to supersede ethical considerations for the implementation of ATT. A systematic review by Teipel et al. in 2016 regarding ATT solutions for navigation purposes for PwD, recommends a clear distinction between safety and autonomy and suggests that future technologies should be better able to assess safety features of the environment and the PwD [66]. Hine et al. (2022) explored ethical considerations in the design and implementation of home-based smart care for dementia in a review using a case study from the National Healthcare System in the United Kingdom [65]. They recommend to design ethics into smart healthcare concepts using a human-centered design, an intersection of various frameworks as guidance, and a network of multi-disciplinary stakeholders as advisers [65].

### Timeliness

Responsibility for timeliness of ATT implementation falls to healthcare and municipality representatives alike, and on multiple tiers of the healthcare ecosystem. The included study by Holthe et al. [45] found that the provision of ATT took an average of 7.5 weeks within the study. This should be “food for thought” considering the progressive nature of dementia and the stage in which introduction to ATT is usually made. Introduction to viable options for ATT should be made at the earliest possible opportunity to fully realize the potential and usefulness of these novel solutions, rather than in crisis or post-crisis situations. This means that levels of healthcare which are involved in making early diagnosis and providing support care must be educated on the benefits and availability of ATT for PwD and informal caregivers. In addition, goals for habitual use should include continual evaluation and tailoring of the interventions. Guisado-Fernandez et al. (2019) conducted a scoping review and design framework looking at factors influencing the adoption of smart health technologies for PwD and their formal and informal caregivers [69]. One theme they discuss is condition-related challenges, including appropriate timing for implementation of technology and how the degree of decline (disease progression) effects participation and use [69]. Factors that promoted use included unobtrusiveness, ease of use, familiarity, intuitiveness, use of common language, planned onboarding and support, sensory, motricity and durability [69].

### Cultural relevance

Cultural relevance is an important consideration when conceptualizing the potential generalization of results from these often smaller and diverse studies, and from a high-income country to LMIC. Although direct generalization in most cases is not possible, the conceptual knowledge of specific promotors and barriers which influence implementation and adoption of ATT globally, can essentially be viewed as core elements and guidance strategies. Necessary adaptation surrounding cultural contexts should be applied when developing future strategies for implementation. Considering the amount of immigration and refugee seekers globally over the last decade this concept will become increasingly relevant in LMIC and high-income countries alike. Although not directly addressed in the included literature, fear, shame, stereotypes, and prejudices are some of the emerging themes found in recent studies regarding cultural stigmas surrounding the diagnosis of dementia [70, 71]. For example, a study conducted in the United Kingdom investigating stigma among primarily Black African and Caribbean communities found that there was a general perception that dementia was a “white person’s illness” [72]. A systematic review by Brooke and Ojo (2020) revealed that there is a common belief in Sub-Saharan Africa that PwD are witches, resulting in abuses and improper care [73]. African American and Latino populations in the USA consistently show higher risk rates for MCI and AD and it is theorized that cultural aspects such as ethnicity, language, country of origin, immigration status, acculturation and healthcare disparities can be associated with these higher rates [74].

Clearly, the complexities of culture and migration globally should be considered when developing implementation strategies and novel education for ATT for PwD within ethnically diverse communities. Improving programs aimed at digital and dementia literacy could empower PwD and formal and informal caregivers and assist in decreasing global stigma surrounding the disease. Another point which is related to improved knowledge and culture is that the “hesitancy to prescribe” concept depicted by Dai et al. may well be in play within varied cultural contexts where knowledge of ATT and its benefits is generally limited [29]. Dai et al. found that formal caregivers were hesitant to make recommendations for ATT due to a lack of knowledge about what was available and how it could ultimately benefit the recipient [29]. This would in theory mean that socio-economic level would play a lesser role in these contexts, meaning that this “hesitancy to prescribe” phenomenon presents equally in middle-to-high income countries and LMIC. Should digital and dementia literacy be improved, you could hypothesize that the desired end result of increased adoption should follow. Further studies are needed to investigate this concept in varied economic and cultural settings taking into consideration certain confounding factors such as overall access to ATT and connectivity (WIFI).

One cultural aspect that has historically been linked to health status is socioeconomic status (SES) [75]. This raises a question for future research as to the association of SES and the effectiveness of ATT implementation and adoption. Typically, lower SES translates to higher mortality and lower health perception. Inherently, there may be a socioeconomic divide within provision of ATT as it is often costly, and recommendations are reliant on access levels within healthcare systems. Therefore, SES can be seen as a potential barrier to provision of ATT. High income countries can be equally as effected as LMICs because there are often large differences in SES within varied ethnic groups [74]. A qualitative study conducted in the Netherlands (2022) by Eggink et al. looking at adults > 55 years (*n* = 19) with low SES concluded that eHealth interventions could be a benefit to improved access to healthcare and lifestyle changes [76]. This point may be at best utopian thinking however and further exploration is needed regarding feasibility, equity, and affordability of such ATT within low SES groups.

### Improved implementation strategies

Powell et al. investigated implementation strategies in healthcare and describes the need for better understanding of barriers and facilitators to trigger future behaviors and better adoption in PwD [62]. The study found that 5 priorities should be established to achieve this goal. They are (1) enhance methods for designing and tailoring implementation strategies (mapping), (2) specify and test mechanisms of change, (3) conduct more effectiveness research on discrete, multi-faceted, and tailored implementation strategies, (4) increase economic evaluations of implementation strategies, and (5) improve tracking and reporting of implementation strategies [62].

### Additional findings

#### Strategic alliances

Strategic collaboration between public and private entities is essential in pushing the development of innovation towards a market ready product [77]. These collaborations may be forged between unlikely partners in the future and could include avenues such as private health insurance providers, industry corporate giants, banks, influencers (social media) and private investors with humanitarian interests. The usual stakeholders should also have a financial interest in the development and forging of market ready ATT for communities. These include government level leadership, universities, municipalities, and healthcare systems [33, 35, 37, 40–42, 46, 59]. Leadership should prioritize strategic alliances with private partners. This could create more opportunity for development and implementation of ATT within communities.

Once an ATT product is ready for the market, the expense of these items directly affects the implementation and adoption choices of PwD and their informal caregivers. Some specific suggestions to assist with implementation and adoption of market ready ATT from informal caregivers within the literature included: government assistance, low interest loans, leasing options, subsidized costs, and complimentary basic support [28, 29, 36, 37, 41, 42, 53, 57]. The idea of a “mixed-economy” approach to service provision was suggested, meaning that state funded social care and private individuals fund ATT provision [41]. This model could be set on a need basis regarding resources of the PwD and the family. More creative options are needed to promote implementation and adoption in this arene. Value, trust, and worthiness of the ATT intervention is often determined by the fidelity and has a significant impact on adoption. With regards to the implementation of new technology we also see that this definition includes the use of the ATT for other intended purposes. For the purposes for this review, we are defining this as *contamination.*

The existing healthcare ecosystem, relying on external service providers for technology design, support and provided competence, is not a sustainable model [28, 33, 35, 36]. In the future, more advanced technology competence must be integrated directly at the municipality and healthcare system levels. An established timeframe for this transition should be considered, combined with co-creation activities between stakeholders. Learning must occur with and between stakeholders at various levels in the ecosystem. Resource integration is an important part of the larger process towards sustainability. Sharing of knowledge, tools and other resources should occur from the top levels to the end-users. This model can assist with a “shared-economy” approach and offer the end-users support throughout the implementation process [41].

#### Implementation frameworks & theories

The success of emerging and future research can be promoted by using current frameworks and theories. These are important contributions and guidelines that can assist future researchers and implementers in efforts to bridge gaps between research and real-world use of ATT for PwD. Just four of the thirty included studies in this review utilized the assistance of an implementation framework or theory, and very few provided a quality description of implementation strategies used [29, 34, 36, 59]. The included frameworks within the review were: United Theory of Acceptance and Use of Technology (UTAUT), Measurement Instrument for Determinants of Innovation (MIDI), Twigg and Atkin’s typology, and the Medical Research Council (MRC) framework [46, 71–73].

Implementation Science is an emerging field of study which focuses on the *research-to-practice gaps* that have unfortunately been very prominent and often criticized in recent years. Bauer defines Implementation Science as “the scientific study of methods to promote the systematic uptake of research findings and other evidence-based practices into routine practice, and, hence, to improve the quality and effectiveness of health services” [78]. Implementation research outcomes may include topics such as acceptability, adoption, appropriateness, feasibility, fidelity, implementation costs, coverage, and sustainability [79]. Implementation can notably be influenced by external complex factors such as implementation strategies by investors which may compromise the effectiveness of the intervention [80]. Researchers must therefore be prepared to challenge decision makers to ensure a balance between compromises made and must address the important topics of fidelity (delivery as originally designed) and adaptation by identifying core and discretionary components of their interventions [80]. It appears the most effective implementation studies utilize a variety of combined frameworks and theories in order to include important elements such as factoring for complexity of intervention (or disease), maintenance of implementation, evaluation, context, scale-out and scale-up, adaptation, identification of core and discretionary components, social validity, fidelity, drift, replication and follow up [81].

One framework suggestion for future studies we would like to highlight as an example for the purpose of this review is “The Promoting Action on Research Implementation in Health Services”, or PARIHS framework. Harvey and Kitson describe the evolution of the PARIHS framework to the now revised I-PARIHS framework and state that it “was developed in an attempt to represent the dynamic and multi-faceted nature of implementation in healthcare” [82]. The main construct of the now I-PARIHS framework is the use of a facilitator(s) as the “active ingredient” of implementation, driving the implementation efforts, applying, and revising strategies, engaging relationships with stakeholders, and negotiating barriers within a contextual setting.

The idea of the healthcare worker and/or the caregiver as the facilitator(s) of ATT implementation could provide a working model at the municipality level for better uptake of innovation and eventual desired result of adoption of new technology. In addition, a framework such as RE-AIM could be combined to assess the elements of maintenance and evaluation missing from the I-PARIHS framework: Reach, Effectiveness, Adoption (setting and staff), Implementation and Maintenance (individual and setting). RE-AIM is widely used across diverse study designs and is easily adaptable [83]. Although we highlight I-PARIHS and RE-AIM, it is important to keep in mind that there are many available resources in the field of Implementation Science that can be utilized for future studies in efforts to strengthen study design and address research-to-practice gaps surrounding implementation and adoption of ATT for PwD and their formal and informal caregivers.

#### Concept of contamination

An interesting finding was something that was referred to in the literature as “bricolage” which references a “do it yourself” strategy for implementation of ATT. Greenhalgh et al. (2013) said a ‘bricoleur’ is: a person who was open and knowledgeable about technologies and who could integrate them into care [61]. We are further defining this however as “contamination” referring to a reference from *Components of Process Evaluation*, and meaning that it is an evaluation of the use of something other than the intended intervention or use of the intervention for unintended purposes (i.e.: prescribed ATT) [84]. This seems to be an emerging strategy to obtain ATT quickly, affordably, and tailor-designed to meet personalized needs [35, 41, 42, 45]. This trend highlights the need for more comprehensive and standardized programs at the municipality and/or public healthcare levels to include a variety of quality ATT providers and sustainable solutions for tailoring, co-design, and of utmost importance, the inclusion of the PwD and the caregiver within the lifespan of the process.

### Limitations of the study

Potential limitations include the potential of missed studies, small study bias, missed outcomes, and compromised detection of missed information. Selective reporting bias and study publication bias can occur which can alter or influence the reported results from the study [25]. The absence of information can affect the overall validity of the review. Included smaller studies may yield a larger than realist estimate of the effect [25]. A limitation of meta-synthesis is that the information is analyzed solely based on the quality assigned to the included articles and there is no “gold standard” for assessment. A final limitation of meta-synthesis is that the thematic analysis of data is subjective, based on the authors background and understanding of the topic. To reduce bias two collaborators were involved in the synthesis and convergent interpretation of the results, the author has utilized CASP, and included thorough analysis of thematic topics identified within the literature, bringing the focus of the review back to the original aim and research questions. A meta-analysis was not performed as the included quantitative literature (*n* = 7) was clinically heterogeneous and used inconsistent specific measurements and metrics.

As mentioned in the results, 94% of the included publications are from high-income countries. We consider this a limitation as it decreases the generalization of the findings and makes conclusions less applicable to LMICs. We do however provide the reader suggestions for use of these findings in high-income countries as core strategies which should be adapted within context to other settings such as LMICs.

## Conclusion

The most crucial elements for the adoption of ATT in the future will be a focus on co-design, improved involvement of both the PwD and their formal and informal caregivers, and the adaptability (tailoring related to context) of ATT solutions over time (disease process). There is a significant need for more quality research to be conducted in the regions of the world where population growth and prevalence of dementia is expected to grow most rapidly over the next 30 years. A global, multi-national implementation guideline should be developed to address these gaps and encompass the complexities of implementation both in high and LMICs.

## Supplementary Information

**Additional file 1.**

**Additional file 2.**

**Additional file 3.**

## Data Availability

Data used in this study is available through the cited journal articles.

## Abbreviations

ATT: Assistive Technology and Telecare
BPSD: Behavioral and Psychological Symptoms of Dementia
CASP: Critical Appraisal Skills Programme
CDR: Clinical Dementia Rating scale
FAST: Functional Assessment Staging Tool
IoT: Internet of Things
LMIC: Low-and Middle-Income Countries
MIDI: Measurement Instrument for Determinants of Innovation
MMAT: Mixed Methods Assessment Tool
MMSE: Mini Mental Status Evaluation
MRC: Medical Research Counsel framework
PARIHS: The Promoting Action on Research Implementation in Health Services
PRISMA: Preferred Reporting Items for Systematic Reviews and Meta-Analyses
PwD: People with Dementia
RE-AIM: Reach, Effectiveness, Adoption, Implementation and Maintenance
SARs: Social Assist Robots
SES: Socioeconomic status
UTAUT: Unified Theory of Acceptance and Use of Technology
WHO: World Health Organization
