# Supplementary material for: Promotors and barriers to the implementation and adoption of assistive technology and telecare for people with dementia and their caregivers: a systematic review of the literature
Source: BMC Health Serv Res. 2022 Dec 23;22:1573. doi: 10.1186/s12913-022-08968-2 (PMC9780101; doi:10.1186/s12913-022-08968-2)
Supplement: Supplementary file 1 — Additional file 1. [file 12913_2022_8968_MOESM1_ESM.docx]

CASP Quality analysis quantitative literature

N= 7; standard deviation: 0.10; mean: 0.64

CASP Quality analysis Qualitative Literature

N= 19; standard deviation: 0.16; mean: 0.80

MMAT Quality analysis Mixed methods literature

N=4; standard deviation: 0.21; mean: 0.63
