# Supplementary material for: Promotors and barriers to the implementation and adoption of assistive technology and telecare for people with dementia and their caregivers: a systematic review of the literature
Source: BMC Health Serv Res. 2022 Dec 23;22:1573. doi: 10.1186/s12913-022-08968-2 (PMC9780101; doi:10.1186/s12913-022-08968-2)
Supplement: Supplementary file 2 — Additional file 2. [file 12913_2022_8968_MOESM2_ESM.docx]

**Search strategy**

Medline Ovid, APA PsycINFO, EMBASE: February 17, 2021 search terms

| 1. dementia.mp. [mp=title, abstract, heading word, table of contents, key concepts, original title, tests & measures, mesh] |
| --- |
| 2. dementia.m_titl. |
| 3. "alzheimer*".m_titl. |
| 4. cognitive decline.m_titl. |
| 5. lewy body disease.m_titl. |
| 6. neurocognitive disorder.m_titl. |
| 7. telemedicine.mp. [mp=title, abstract, heading word, table of contents, key concepts, original title, tests & measures, mesh] |
| 8. telemedicine.m_titl. |
| 9. "assistive technolog*".m_titl. |
| 10. telecare.m_titl. |
| 11. "smart home*".m_titl. |
| 12. telehealthcare.m_titl. |
| 13. "robotic*".m_titl. |
| 14. "voice technolog*".m_titl. |
| 15. "smart phone*".m_titl. |
| 16. "wearable*".m_titl. |
| 17. "gerontechnolog*".m_titl. |
| 18. web-based.m_titl. |
| 19. digital.m_titl. |
| 20. "sensor*".m_titl. |
| 21. telehealth.m_titl. |
| 22. ehealth.m_titl. |
| 23. telerehabilitation.m_titl. |
| 24. "implement*".m_titl. |
| 25. "barrier*".m_titl. |
| 26. "promot*".m_titl. |
| 27. "facilitat*".m_titl. |
| 28. installation.m_titl. |
| 29. usage.m_titl. |
| 30. "motivat*".m_titl. |
| 31. 1 or 2 or 3 or 4 or 5 or 6 |
| 32. "telemonitor*".m_titl. |
| 33. 7 or 8 or 9 or 10 or 11 or 12 or 13 or 14 or 15 or 16 or 17 or 18 or 19 or 20 or 21 or 22 or 23 or 32 |
| 34. 24 or 25 or 26 or 27 or 28 or 29 or 30 |
| 35. 31 and 33 and 34 |
