# Supplementary material for: Promotors and barriers to the implementation and adoption of assistive technology and telecare for people with dementia and their caregivers: a systematic review of the literature
Source: BMC Health Serv Res. 2022 Dec 23;22:1573. doi: 10.1186/s12913-022-08968-2 (PMC9780101; doi:10.1186/s12913-022-08968-2)
Supplement: Supplementary file 3 — Additional file 3. [file 12913_2022_8968_MOESM3_ESM.docx]

CASP questions for quantitative studies (n=7)

|  | **Yes** | **Can't Tell** | **No** |
| --- | --- | --- | --- |
| Did the study address a clearly focused research question? | 7 |  |  |
| Was the assignment of participants to interventions randomized? | 1 |  | 6 |
| Were all participants who entered the study accounted for at its conclusion? | 6 | 1 |  |
| Were the participants [investigators? People assessing/analyzing outcomes] blind to the intervention? |  |  | 7 |
| Were the study groups similar at the start of the randomized controlled trial? | 7 |  |  |
| Apart from the experimental intervention, did each study group receive the same level of care? | 3 | 4 |  |
| Were the effects of the intervention reported comprehensively? | 7 |  |  |
| Was the precision of the estimate of the intervention or treatment effect reported? | 7 |  |  |
| Do the benefits of the experimental intervention outweigh the harms and costs? | 6 | 1 |  |
| Can the results be applied to your local population/in your context? | 3 | 4 |  |
| Would the experimental intervention provide greater value to the people in your care than any of the existing interventions? | 2 | 5 |  |
|  |  |  |  |
| CASP questions for qualitative studies (n=19) |  |  |  |

|  | **Yes** | **Can’t tell** | **No** |
| --- | --- | --- | --- |
| Was there a clear statement of the aims of the research? | 19 |  |  |
| Is a qualitative methodology appropriate? | 18 | 1 |  |
| Was the research design appropriate to address the aims of the research? | 16 | 3 |  |
| Was the recruitment strategy appropriate to the aims of the research? | 16 | 3 |  |
| Was the data collected in a way that addressed the research issue? | 14 | 5 |  |
| Has the relationship between researcher and participants been adequately considered? | 3 | 16 |  |
| Have ethical issues been taken into consideration? | 18 | 1 |  |
| Was the data analysis sufficiently rigorous? | 16 | 3 |  |
| Is there a clear statement of findings? | 19 |  |  |
| How valuable is the research? | 14 | 5 |  |

Mixed Methods Appraisal Tool (MMAT) questions for mixed methods studies (n=4)

| **MMAT** | **Mixed-Methods Quality Assessment** | **Yes** | **Can’t tell** | **No** |
| --- | --- | --- | --- | --- |
| Screening | Are there clear research questions? Do the collected data allow to address the research questions? | 4 3 |  | 1 |
| Qualitative | Is the qualitative approach appropriate to answer the research question? Are the qualitative data collection methods adequate to address the research question? Are the findings adequately derived from the data? Is the interpretation of results sufficiently substantiated by data? Is there conherence between qualitative data sources, collection, analysis and interpretation? | 4 4 3 1 1 | 1 3 3 |  |
| Quantitative non-randomized | Are the participants representative of the target population? Are measurements appropriate regarding both outcome and intervention? Are there complete outcome data? Are the confounders accounted for in the design and analysis? During the study period, is the intervention administed as intended? | 3 2 2  3 | 1  2 | 1 1 |
| Quantitative  descriptive | Is the sampling strategy relevant to address the research question? Is the sample representative of the target population? Are the measurements appropriate? Is the risk of nonresponse bias low? Is the statistical analysis appropriate to answer the research question? | 1 1 1  1 | 1 |  |
| Mixed Methods | Is there an adequate rationale for using a mixed methods design to address the question? Are the different components of the study effectively integrated to answer the question? Are the outputs of the integration of qualitative and quantitative components adequately interpretated? Are divergences and inconsistencies between quantitative and qualitative results adequately addressed? Do the different components of the study adhere to the quality criteria of each tradition of the methods involved? | 4 2 1  1  3 | 2 3  3  1 |  |
